# Benzophenones from *Anemarrhena asphodeloides* Bge. Exhibit Anticancer Activity in HepG2 Cells via the NF-κB Signaling Pathway

**DOI:** 10.3390/molecules24122246

**Published:** 2019-06-15

**Authors:** De-Ling Wu, Zhen-Dong Liao, Fang-Fang Chen, Wei Zhang, Ya-Shuo Ren, Can-Can Wang, Xiao-Xiao Chen, Dai-Yin Peng, Ling-Yi Kong

**Affiliations:** 1 Jiangsu Key Laboratory of Bioactive Natural Product Research and State Key Laboratory of Natural Medicines, School of Traditional Chinese Pharmacy, China Pharmaceutical University, 24 Tong Jia Xiang, Nanjing 210009, China; dlwu7375@sina.com; 2 School of Pharmacy, Anhui University of Chinese Medicine, Hefei 230012, China; yyfhzwz@163.com (Z.-D.L.); rtsjxfc@163.com (F.-F.C.); zhangwei@ahtcm.edu.cn (W.Z.); renyashuo@gmail.com (Y.-S.R.); cancanW1120@163.com (C.-C.W.); 15077919652@163.com (X.-X.C.); 3 Anhui Province Key Laboratory of Traditional Chinese Medicine Decoction Pieces of New Manufacturing Technology, Anhui University of Chinese Medicine, Hefei 230012, China

**Keywords:** *Anemarrhena asphodeloides* Bge., benzophenones, hepatocellular carcinoma, apoptosis, nuclear factor-κB

## Abstract

A chemical investigation of the fibrous roots of *Anemarrhena asphodeloides* Bge. led to the isolation of four benzophenones, including one new compound (**1**) and three known ones (**2–4**). Comprehensive ^1^D, ^2^D NMR and HRESIMS data established the structures of the isolated compounds. The absolute configurations were determined by comparison of the calculated optical rotation (OR) with experimental data. All the isolates were evaluated for their cytotoxicities on hepatocellular carcinoma cell lines (HepG2 and Hep3B). Compound **1** showed strong cytotoxicity against HepG2 and Hep3B cells, with IC_50_ values at 153.1 and 180.6 nM. Through MTT assay, flow cytometry and Western blot analysis, compound **1** demonstrated the ability to stimulate apoptosis via the NF-κB signaling pathway in HepG2 cells. These benzophenones are potential lead compounds for the development of better treatments for hepatocellular carcinoma.

## 1. Introduction

Hepatocellular carcinoma (HCC) is one of the most common malignancies worldwide that poses a great menace to human health and life. As one of the most lethal tumors, HCC is intractable with no effective treatment [1]. Various signaling pathways are dysregulated during the pathogenesis of HCC [2]. In HCC tumor tissues, the activation of nuclear factor-κB (NF-κB) has frequently been observed. NF-κB regulates the growth of HCC [3], and plays an important role in tumorigenesis [4]. The deficiency of NF-κB regulators also results in spontaneous liver injury, fibrosis and HCC [5,6]. Moreover, the activation of NF-κB could promote the production of cytokines and chemokines, leading to the development of HCC [7].

Natural products play essential roles in the development of anticancer drugs [8,9,10,11]. *A. asphodeloides* Bge., a famous traditional Chinese medicine, has been used as an antipyretic, anti-inflammatory and antiplatelet aggregator [12]. An increasing amount of evidence has revealed the potential antitumor activity of *A. asphodeloides* Bge. [13,14]. As the main active components of *A. asphodeloides* Bge., benzophenones have a broad spectrum of biological and pharmacological activities [15,16].

**Figure 1:**
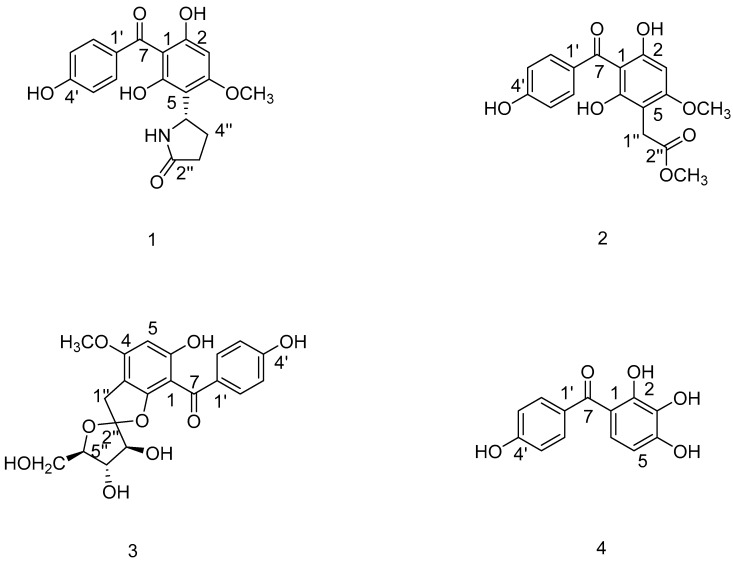
The structures of compounds **1**–**4**.

The biological activity results have prompted us to continuously study the benzophenone components of *A. asphodeloides* Bge. This study described the isolation and elucidation of four benzophenones (**1**–**4**) (Figure 1) and their cytotoxicities against two HCC cell lines (HepG2 and Hep3B). In addition, we investigated whether compound **1** could induce the apoptosis of HepG2 cells and regulate the NF-κB signaling pathway.

## 2. Results 

### 2.1. Identification of Compounds ***1**–**4*** from the Fibrous Roots of A. asphodeloides Bge.

Compound **1** was obtained as a green amorphous powder. It had a molecular formula of C_18_H_17_NO_6_ with eleven degrees of unsaturation, based on the high resolution electrospray ionization mass spectroscopy (HRESIMS) at *m*/*z* 366.0947 [M + Na]^+^ (calculated for C_18_H_17_NO_6_Na, 366.0947), which were further verified by a ^13^C NMR spectrum. The ^1^H NMR spectrum presented five aromatic proton signals at δ_H_ 7.53 (2H, d, *J* = 8.7 Hz, H-2′, -6′), δ_H_ 6.78 (2H, d, *J* = 8.7 Hz, H-3′, -5′) and δ_H_ 6.08 (1H, s, H-3), and also allowed the identification of two methylene groups (δ_H_ 2.32 (1H, m, H-3″), δ_H_ 2.28 (1H, m, H-4″), δ_H_ 2.16 (1H, m, H-3″) and δ_H_ 2.01 (1H, m, H-4″)), one methoxyl proton signal at δ_H_ 3.74 (3H, s, 4-OCH_3_) and one methyne proton signal at δ_H_ 5.11 (1H, d, *J* = 4.9, 9.3Hz, H-5″). The analysis of the ^13^C NMR spectra (Table 1), in combination with Distortionless Enhancement by Polarization Transfer (DEPT) experiments, revealed the presence of eighteen carbon signals, including twelve aromatic carbons, combined with the carbonyl signal at δ_C_ 195.8 (C-7). These features indicated that compound **1** was a typical benzophenone derivative [17].

**Figure 2:**
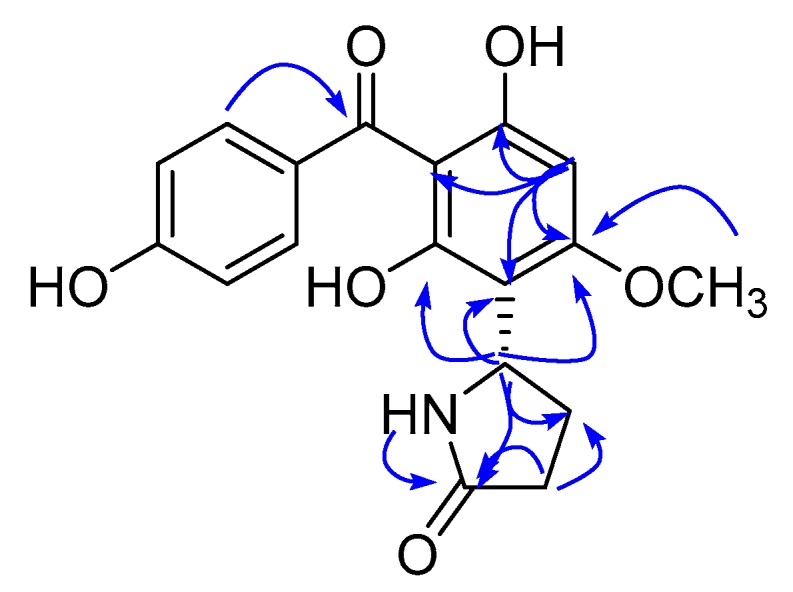
Key HMBC correlations of compound **1**.

A pyrrolidone moiety was established by the HMBC correlations (Figure 2) of H-5″ at δ_H_ 5.11 with C-2″ and C-4″, and of H-3″ with C-2″ and C-4″. The HMBC correlations from H-5″ to C-4′, C-5′ and C-6′, and from H-4″ to C-5′ allowed the attachment of the 2-oxopyrrolidin-5yl group at C-5′. The absolute configuration of compound **1** was determined by comparison of its experimental and calculated optical rotation (OR) spectra with data from literature [18]. On the basis of the comparison, the absolute configuration of compound **1** (Figure 2) was assigned as (*S*)-5-(2,4-dihydroxy-3-(4-hydroxybenzoyl)-6-methoxyphenyl)pyrrolidin-2-one. The known benzophenones were identified as Methyl 2-(2,4-dihydroxy-3-(4-hydroxybenzoyl)-6-methoxyphenyl)acetate (**2**) [19], 4′,6-dihydroxy-4-methoxybenzophenone-2-*O*-(2″),3-C-(1″)-1″-desoxy-*α*-l-fructofuranoside (**3**) [20], and (4-hydroxyphenyl)(2,3,4-trihydroxyphenyl)methanone (**4**) [21] by analyzing their spectral data and comparing them with reported literature values.

### 2.2. Cytotoxicity In Vitro

An analysis of cell viability using an MTT assay showed that compounds **1**–**4** were able to inhibit cell viability significantly (*p* < 0.01). In particular, compound **1** displayed the most potent cytotoxicity, with an IC_50_ value of 153.10 ± 34.30 nM (Table S1, Supplementary Materials). The results warranted further investigation of compound **1** for the underlying mechanism in HepG2 cells.

### 2.3. Apoptosis Study

**Figure 3:**
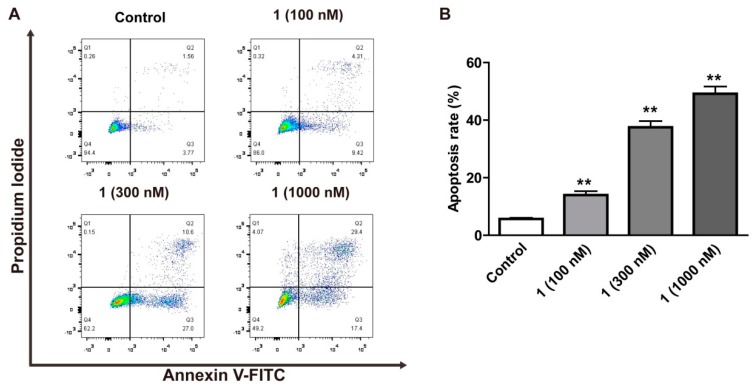
The effect of compound **1** on apoptosis in HepG2 cells. (**A**,**B**) Cells stained by annexin V-FITC and PI were measured using flow cytometry. The results were presented as mean ± SD (*n* = 3). Compared with the control group, ** *p* < 0.01.

**Figure 4:**
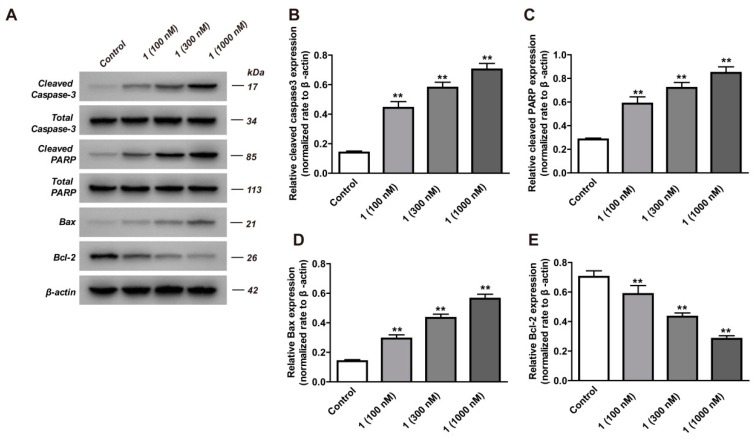
The effects of compound **1** on cleaved caspase-3, caspase-3, cleaved PARP, total PARP, Bax, Bcl-2 expressions in HepG2 cells. HepG2 cells were cultured in the presence or absence of compound **1** (100, 300, 1000 nM) for 24 h. (**A**) The expressions of cleaved caspase-3, caspase-3, cleaved PARP, total PARP, Bax and Bcl-2 were measured by Western blot assay, with *β*-actin serving as a loading control. (**B**–**E**) The relative expressions of cleaved caspase-3, caspase-3, cleaved PARP, total PARP, Bax and Bcl-2 were normalized to *β*-actin. Results are shown as mean ± SD for the three individual experiments which, for each condition, were performed in triplicate. Compared with the control group, ** *p* < 0.01.

To investigate whether compound **1** could induce apoptosis in HepG2 cells, flow cytometry was used to detect the percentage of apoptotic cells. As shown in Figure 3A,B, compound **1** (100, 300, 1000 nM) can promote the apoptosis rate from 5.68% to 13.97%, 37.54% and 49.19% in a dose-dependent manner. In addition, we evaluated the level of apoptotic proteins using Western blot analysis. The results showed that compound **1** was able to upregulate the expression of the proapoptotic protein Bax but downregulated the expression of antiapoptotic protein Bcl-2 (Figure 4A,D,E). Caspase-3 can be activated by upstream apoptotic signaling, which cleaves various cellular substrates such as poly ADP-ribose polymerase(PARP) [22]. Furthermore, this study showed that the expressions of cleaved caspase-3 and cleaved PARP were significantly enhanced in compound **1**-treated HepG2 cells (Figure 4A–C).

### 2.4. Inhibition Analysis of NF-κB

**Figure 5:**
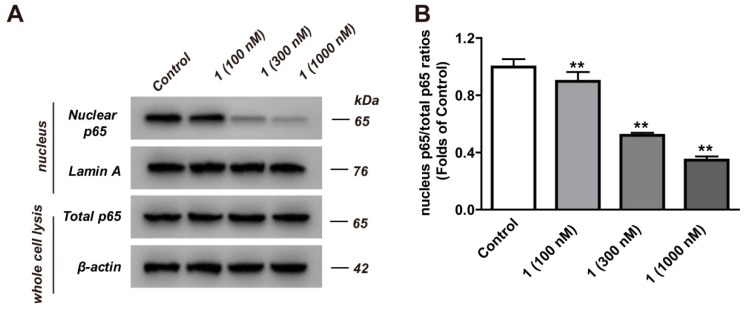
The effects of compound **1** on p65 nuclear translocation in HepG2 cells. HepG2 cells were cultured in the presence or absence of compound **1** (100, 300, 1000 nM) for 24 h. (**A**) The expression of total p65 and nuclear p65 were measured by Western blot assay, with β-actin or Lamin A serving as a loading control. (**B**) The results were normalized to the control group. Results are shown as mean ± SD for three individual experiments which, for each condition, were performed in triplicate. Compared with the control group, ** *p* < 0.01.

NF-κB activation is a frequent event in HCC [23]. The expression of NF-κB p65 in liver cancer tissues is higher than other tissues, and the key to regulating NF-κB function is to inhibit the promotion of p65 in liver tumors [24]. In this study, compound **1** was able to downregulate the expression of nuclear p65 in a dose-dependent manner (Figure 5A,B). These results implied that compound **1** was able to promote the occurrence of apoptosis by inhibiting the nuclear translocation of p65. Collectively, these findings indicated that compound **1** was able to exert this effect by regulating the NF-κB pathway in HepG2 cells.

## 3. Discussion

Approximately 700,000 people die of HCC each year worldwide. As the fifth most frequent cancer across the world, HCC has high prevalence rates due to hepatitis B virus infection [25]. Only around 30% of patients with HCC are suitable to treat with surgical resection and liver transplantation [26]. Lacking clear symptoms and overlapping with other diseases, only 25% of HCC patients are diagnosed within early stage HCC [27,28]. Treatment options of HCC include surgical resection, ablation, transarterial chemoemobolization, liver transplantation, chemotherapy and radiotherapy [29]. However, no single treatment has achieved the desired results. More and more natural products have provided health benefits to humans [30]. Historically, virtually all medicinal preparations came from natural products, i.e., from plants and animals [31]. Natural products play essential roles in the development of anticancer drugs.

In the present study, four benzophenone compounds (**1**–**4**) were isolated and identified from the fibrous roots of *A*. *asphodeloides* Bge. (*S*)-5-(2,4-dihydroxy-3-(4-hydroxybenzoyl)-6-methoxyphenyl)pyrrolidin-2-one (**1**) was a new compound and the know benzophenones (**2**–**4**) were first reported in *Anemarrhena* Bunge. Benzophenone compounds are found in natural plants, with special structures existing in Iridaceae, Myrtle, Liliaceae, Garcinia, Rosaceae, Lauraceae, Moraceae and other plants. These compounds are distributed in leaves, stems, flowers, roots, fruits and other parts of plants. The basic skeleton, containing thirteen carbon atoms, is composed of two benzene rings connected by a carbonyl group. More and more evidence [32,33] has shown that many natural benzophenones are cytotoxic on HCC cell lines, so we evaluated the cytotoxicity of compounds (**1**–**4**) on HCC cell lines (HepG2 and Hep3B). We were pleasantly surprised to find that compound **1** showed strong cytotoxicity against HepG2 cells, with an IC_50_ value of 153.1 nM.

Apoptosis is a pivotal physiological process for the normal development and maintenance of tissue homeostasis [34]. Because the importance of apoptosis regulation is a key factor in the development of anticancer drugs, this pattern of death has been extensively studied. NF-κB is a significant transcription factor that regulates hepatic homeostasis by regulating the expression of genes and molecules. Recently, some reports [10,35] have suggested that the inhibition of the expression of NF-κB could promote apoptosis and achieve anti-hepatocarcinoma effects. p65, encoded by RELA, is one of the five cellular DNA binding subunits of the human NF-κB family. The c-terminus of p65 contains the strongest activating factor at most κB sites [36]. To investigate the effect of NF-κB on HepG2 cells, we used Western blot analysis to investigate the effects of compound **1** on the expression of p65 in HepG2 cells. The experimental data showed that compound **1** was able to downregulate the expression of nuclear p65 and inhibit the nuclear translocation of p65. The nuclear translocation of p65 is associated with apoptosis, which is promoted by inhibiting the nuclear translocation of p65 [37]. 

In the Bcl-2 family, Bax is a proapoptotic protein and Bcl-2 is an antiapoptotic protein [38]. The Bax/Bcl-2 ratio determines the physiological process of HCC. An imbalance between Bax and Bcl-2 proteins may result in the activation of caspase 3 and lead to apoptosis [39]. PARP is the main target of caspase 3 in vivo. When PARP loses enzyme activity, it will accelerate cell instability. In our study, compound **1** prominently induced apoptosis in HepG2 cells by downregulating the expression of the antiapoptotic protein Bcl-2 and upregulating the expression of the proapoptotic protein Bax. In addition, the expressions of caspase-3 and PARP were significantly enhanced in compound **1**-treated HepG2 cells. Our results demonstrated that compound **1** effectively induced apoptosis by inhibiting the NF-κB signaling pathway, suggesting that NF-κB could be an important target for HCC therapy.

## 4. Materials and Methods

### 4.1. General Experimental Procedures

Optical rotation was recorded on a Jasco P-1020 automatic digital polarimeter. The UV spectrum was measured on a Shimadzu UV-2401PC spectrophotometer (Shimadzu, Japan). The IR spectrum was obtained on a Bruker Tensor 27 FT-IR spectrometer (Bruker, Germany) with KBr pellet. NMR spectra were recorded on Bruker AVIII-800 instruments (Bruker, Germany) with Tetramethyl silane (TMS) as an internal standard. HRESIMS experiments were measured by a MicroTOF spectrometer (Bruker, Germany). The chromatographic silica gel (200–300 mesh) was produced by Qingdao Ocean Chemical Factory. Macroporous adsorption resin AB-8 was produced by Cangzhou Bon Adsorber Technology Co., Ltd. (Cangzhou, Heibei, China). Semipreparative RP-HPLC isolation was achieved with a Shimadzu LC-6 AD instrument using a YMC C18 column (250 × 10 mm, 5 μM, Japan).

### 4.2. Plant Material

The fibrous roots of *A. asphodeloides* Bge. were collected in Bozhou, Anhui province, China, in November 2015, and were identified by Professor Shou-Jin Liu (School of Pharmacy, Anhui University of Chinese Medicine). A voucher specimen (No. 20151101) has been deposited in the herbarium of Anhui University of Chinese Medicine, Hefei, China.

### 4.3. Extraction and Isolation

The 70% EtOH extract (3.5 kg) obtained from the dried fibrous roots of *A. asphodeloides* Bge. (30 kg) was partitioned in water and extracted with ethyl acetate. The water layer was concentrated and applied to an AB-8 macroporous resin column eluted with EtOH–H_2_O (10:90 and 90:10, *v*/*v*). The 90% ethanol portion (1.4 kg) was subjected to silica gel column chromatography (CH_2_Cl_2_–MeOH, 100:1–0:100, *v*/*v*) to afford seven fractions (1–7). Fr.2.1–Fr.2.8 from Fr.2 (45 g) were obtained by an octadecylsilyl (ODS) column (sequentially eluted with 30% MeOH, 60% MeOH and 90% MeOH). Fr. 2.7 (0.5 g) was chromatographed on a silica gel column eluted with CH_2_Cl_2_/MeOH (100:1–0:100, *v*/*v*) to give Fr.2.7.1–Fr.2.7.4. Fr.2.7.3 (170 mg) was subjected to preparative column chromatography (MeOH–H_2_O = 48: 52, *v*/*v*, 7 mL·min^−1^) to obtain Fr.2.7.3.1–Fr.2.7.3.4. Fr.2.7.3.4 (61 mg) was used for semi-preparative column chromatography to give compound **1** (23 mg, t_R_ = 59.1 min, CH_3_CN–H_2_O = 26:74, *v*/*v*). Fr.2.8.1.4 (148 mg), Fr.2.6.2.5 (163 mg) and Fr.1.3 (513 mg) were similarly purified by semi-preparative HPLC to obtain **2** (8 mg, t_R_ = 35.3 min, CH_3_CN–H_2_O = 36:64, *v*/*v*), **3** (10 mg, t_R_ = 45.3 min, CH_3_CN–H_2_O = 25:75, *v*/*v*) and **4** (7 mg, t_R_ = 20.3 min, MeOH–H_2_O = 51:49, *v*/*v*), respectively.

### 4.4. Characterization of Compound ***1***

Green amorphous powder. [*α*${]}_{D}^{25}$−172.00 (*c* 0.10, MeOH), [*α*${]}_{D}^{25}$−327.12 (*c* 0.10, MeOH) (calculated OR). UV (MeOH) *λ*_max_ (log *ε*): 194 nm (4.11), 257 nm (3.33), 311 nm (3.64). IR (KBr) *ν*_max_: 3427, 1656, 1607, 1512 cm^−1^. ^1^H NMR and ^13^C NMR data (Table 1). HRESIMS *m*/*z* 399.0954 [M + Na]^+^ (calcd for C_18_H_17_NO_6_Na 366.0947).

### 4.5. Optical Rotation Calculations

The specific optical rotation was calculated by density functional theory (DFT) at the B3LYP/6–311++ G (2d, p) level with the Conductor-like polarizable continuum model (CPCM) solvation model, where MeOH was used as the solvent to match the experimental conditions, and the stated wavelength was 589.3 nm [40].

### 4.6. Cell Culture

HepG2 and Hep3B cells (ATCC, Manassas, VA, USA) were subcultured under the conditions of Eagle’s Minimum Essential Medium (EMEM) containing penicillin (final concentration of 100 U/mL), streptomycin (final concentration of 100 U/mL), and 10% Fetal bovine serum (FBS). When the cells were fused to 90%, the old medium was discarded. The cells were washed twice with 2 mL of Phosphate buffer saline (PBS). After discarding the PBS, 2 mL 0.25% (*w*/*v*) Trypsin-0.53 mM Ethylenediaminetetraacetic acid (EDTA) mixed digest was added and observed under a microscope for about 30 s. When the cells were rounded, 2 mL of complete medium was added quickly to stop digestion, and gently pipetted to collect the cells. After centrifugation at 800 rpm, 4 °C for 5 min, the cells were resuspended in complete medium (changed every other day), and cultured in divided bottles.

### 4.7. MTT Assay

The growth inhibitory effect of different compounds on tumor cells was examined by MTT assay [41]. Cells were seeded on a 96-well plate at a final density of 1 × 10^5^ cells/well and cultured. Subsequently, the supernatant was discarded, and cells were incubated in dulbecco’s modified eagle medium (DMEM) medium containing different concentrations of compounds (10, 100, 1000, 5000 and 10,000 nM) for 72 h. 20 μL of MTT (5 mg/mL) was added to each well. The supernatant of each pore was discarded. Adding 150 μL DMSO to each pore, the supernatant was shaken for 10 min. The OD_570_ value was determined by the enzyme-labeled instrument after crystallization was completely dissolved. The IC_50_ values were calculated by GraphPad Prism 7.0. 5-Fluorouracil (Selleck Chemicals, Houston, TX, USA) was used as a positive control.

### 4.8. Flow Cytometric Analysis

The method of flow cytometry experiment referred to the previous research [10]. HepG2 cells were cultured at a density of 1 × 10^6^ cells per well overnight. After 24 h treatment of compound **1** at different concentrations (100, 300, 1000 nM), at room temperature for 15 min the treated cells were stained with Annexin V-FITC followed by Propidium Iodide (PI). Cell samples were analyzed by Fluorescence activated Cell Sorting (FACS) can flow cytometry (Becton Dickinson, Franklin Lakes, NJ, USA).

### 4.9. Western Blot Analysis

The method of Western blotting referred to the previous research [41]. After treatment with compounds, cells were cultured for 24 h. Treated cells were then lysed in RIPA buffer. Protein concentration in the lysates was determined using a Bicinchoninic acid (BCA) Protein Assay Kit. Proteins (10 μL) were separated by 10% polyacrylamide gel electrophoresis (SDS-PAGE) gels and transferred onto polyvinylidene difluoride membrane. Following 1 h incubation in a fresh PBS buffer, the blots were probed with specific antibodies overnight at 4 °C, and then with the secondary antibody at room temperature for 2 h. Immunolabeling was then visualized by the Electrochemiluminescence (ECL) kit.

### 4.10. Statistical Analysis

Statistical analysis was carried out using SPSS 17.0 software. Results are expressed as mean ± standard deviation. Comparisons between groups were performed by Tukey’s test and one-way ANOVA. *p* < 0.05 was considered statistically significant.

## 5. Conclusions

**Figure 6:**
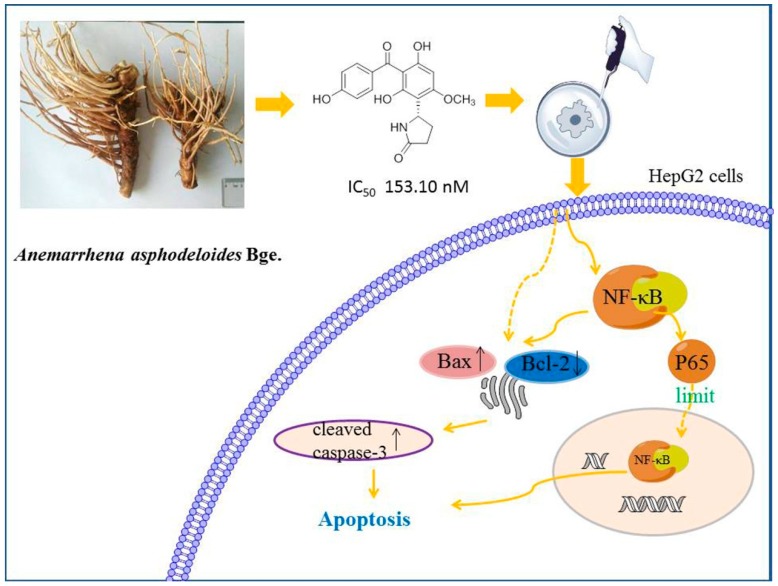
Schematic overview of the proposed mechanism of benzophenones from *Anemarrhena asphodeloides* Bge. in HepG2 cells.

We isolated and identified benzophenones from the fibrous roots of *Anemarrhena asphodeloides* Bge. and tested their cytotoxicity effects on liver cancer cells. Among them, compound **1** has the most significant cytotoxic effect on HepG2 cells. Subsequently, we found that compound **1** could downregulate the pathway of apoptosis in HepG2 cells via the NF-κB pathway. These results indicated that compound **1** may exhibit an anticancer effect in the liver via NF-κB (Figure 6).

## Figures and Tables

**Table 1:** ^1^H NMR and ^13^C NMR spectroscopic data for compound **1** (DMSO-*d*_6_).

Positions	δ_H_ (*J* in Hz)	δ_C_
1	-	106.8
2	-	158.3
3	6.08 (1H, s, H-3)	91.5
4	-	161.5
5	-	108.6
6	-	157.8
7	-	195.8
1′	-	130.8
2′, 6′	7.53 (2H, d, *J* = 8.7 Hz)	131.6
3′, 5′	6.78 (2H, d, *J* = 8.7 Hz)	114.6
4′	-	161.8
1″	-	-
2″	-	176.8
3″	2.32 (1H, m) 2.16 (1H, m)	30.7
4″	2.28 (1H, m) 2.01 (1H, m)	25.8
5″	5.11 (1H, d, *J* = 4.9, 9.3 Hz)	46.9
4-OCH_3_	3.73 (3H, s)	55.5

^1^H NMR spectra were recorded at 800 MHz and ^13^C NMR spectra were recorded at 200 MHz.

## Supplementary Materials

The supplementary materials are available online.
