# Supplementary material for: Benzophenones from Anemarrhena asphodeloides Bge. Exhibit Anticancer Activity in HepG2 Cells via the NF-κB Signaling Pathway
Source: Molecules. 2019 Jun 15;24(12):2246. doi: 10.3390/molecules24122246 (PMC6630431; doi:10.3390/molecules24122246)
Supplement: Supplementary file 1 [file molecules-24-02246-s001.pdf]

SPPLEMENTARY MATERIAL

Article

Benzophenones from *Anemarrhena asphodeloides* Bge. Exhibit Anticancer Activity in HepG2 Cells via the NF-κB Signaling Pathway

De-Ling Wu [1,2,3,†], Zhen-Dong Liao [2,†], Fang-Fang Chen [2], Wei Zhang [2,3], Ya-Shuo Ren [2,3], Can-Can Wang [2], Xiao-Xiao Chen [2], Dai-Yin Peng [2,*] and Ling-Yi Kong [1,*]

[1] Jiangsu Key Laboratory of Bioactive Natural Product Research and State Key Laboratory of Natural Medicines, School of Traditional Chinese Pharmacy, China Pharmaceutical University, 24 Tong Jia Xiang, Nanjing 210009, China; dlwu7375@sina.com

[2] School of Pharmacy, Anhui University of Chinese Medicine, Hefei 230012, China; yyfhzwz@163.com (Z.-D.L.); rtsjxfc@163.com (F.-F.C.); zhangwei@ahtcm.edu.cn (W.Z.); renyashuo@gmail.com (Y.-S.R.); cancanW1120@163.com (C.-C.W.); 15077919652@163.com (X.-X.C.)

[3] Anhui Province Key Laboratory of Traditional Chinese Medicine Decoction Pieces of New Manufacturing Technology, Anhui University of Chinese Medicine, Hefei 230012, China

* Corresponding: pengdaiyin@163.com (D.-Y.P.); cpu_lykong@126.com (L.-Y.K.); Tel.: +86-0551-68129004 (D.-Y.P.); +86-25-83271405 (L.-Y.K.)

† These authors contributed equally to this work

List of supplementary content

Figure S1.1 UV spectrum of compound **1**

Figure S1.2 IR spectrum of compound **1**

Figure S1.3 ^{1}H NMR spectrum (800 MHz, DMSO-d_6) of compound **1**

Figure S1.4 ^{13}C NMR spectrum (200 MHz, DMSO-d_6) of compound **1**

Figure S1.5 DEPT spectrum (200 MHz, DMSO-d_6) of compound **1**

Figure S1.6 HMBC spectrum (800 MHz, DMSO-d_6) of compound **1**

Figure S1.7 HSQC spectrum (800 MHz, DMSO-d_6) of compound **1**

Figure S1.8 HRESIMS spectrum of compound **1**

Figure S1.9 Calculating OR spectra of compound **1**

Table S1 Growth inhibition of HepG2 and Hep3B cells in the presence of different compounds after 72 h incubation (IC50 values in nM).

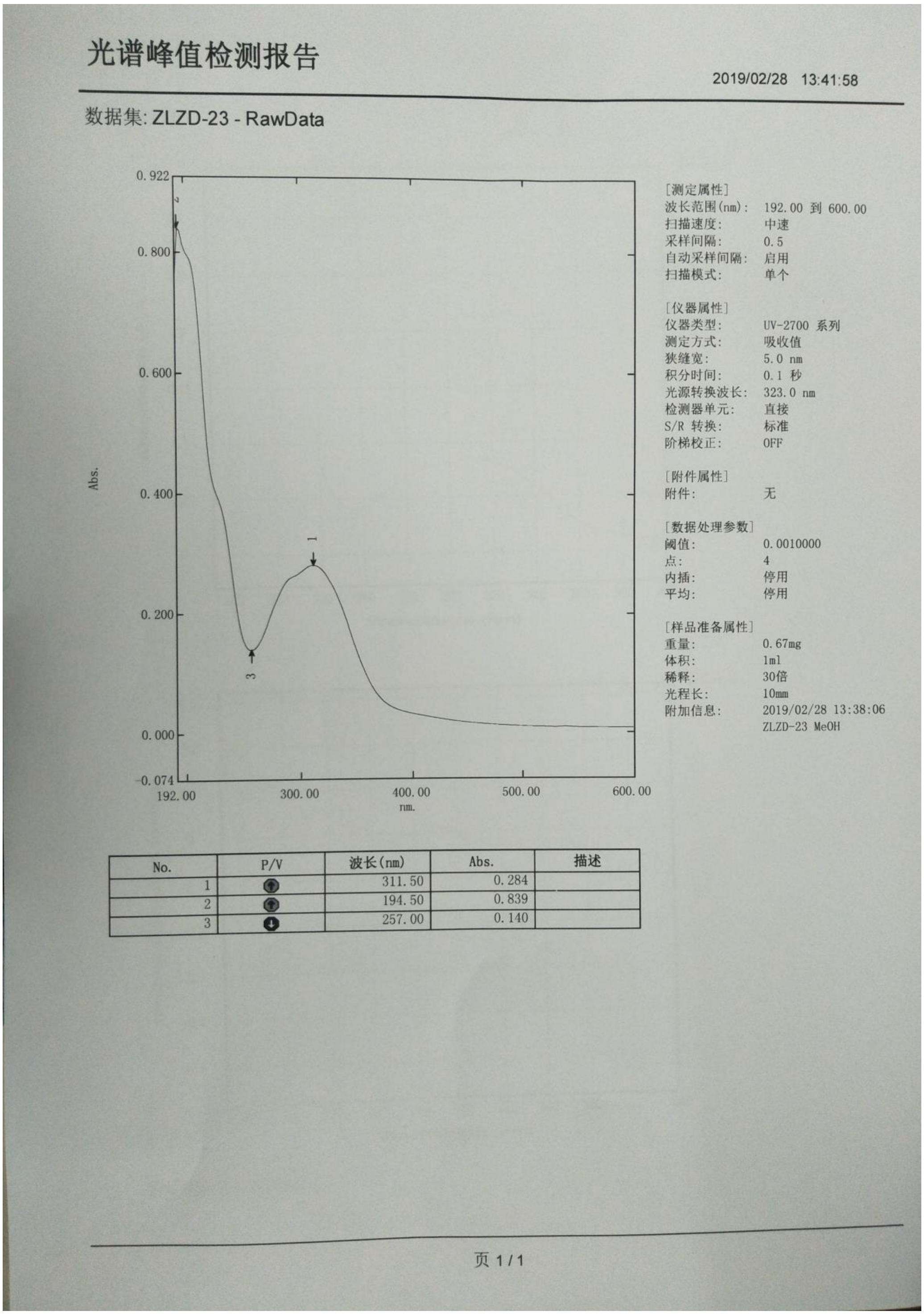

光谱峰值检测报告

2019/02/28 13:41:58

数据集: ZLZD-23 - RawData

[测定属性]
波长范围(nm): 192.00 到 600.00
扫描速度: 中速
采样间隔: 0.5
自动采样间隔: 启用
扫描模式: 单个

[仪器属性]
仪器类型: UV-2700 系列
测定方式: 吸收值
狭缝宽: 5.0 nm
积分时间: 0.1 秒
光源转换波长: 323.0 nm
检测器单元: 直接
S/R 转换: 标准
阶梯校正: OFF

[附件属性]
附件: 无

[数据处理参数]
阈值: 0.0010000
点: 4
内插: 停用
平均: 停用

[样品准备属性]
重量: 0.67mg
体积: 1ml
稀释: 30倍
光程长: 10mm
附加信息: 2019/02/28 13:38:06
ZLZD-23 MeOH

No.	P/V	波长(nm)	Abs.	描述
1	↑	311.50	0.284	
2	↑	194.50	0.839	
3	↓	257.00	0.140	

页 1 / 1

Figure S1.1 UV spectrum of compound **1**

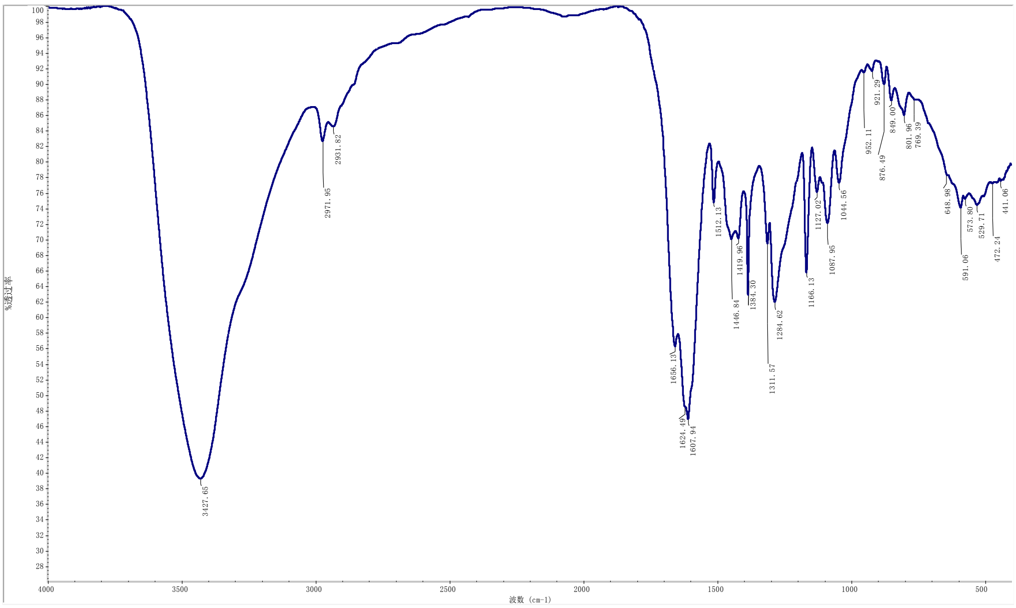

Sample Name: ZLZD-23
KBr压片
采集时间: 星期一 3月 04 15:37:04 2019 (GMT+08:00)
仪器型号: NICOLET iS10
Software version: OMNIC 9.8.372

样品扫描次数: 16
背景扫描次数: 16
分辨率: 4.000
采样增益: 1.0
动镜速度: 0.4747
光阑: 80.00

星期一 3月 04 15:46:25 2019 (GMT+08:00)
标峰:
谱图: ZLZD-23
范围: 4000.00 400.00
（绝对）阈值: 93.933
灵敏度: 99
峰表:

峰位置:	441.06	强度:	77.473
峰位置:	472.24	强度:	77.122
峰位置:	529.71	强度:	74.364
峰位置:	573.80	强度:	75.166
峰位置:	591.06	强度:	74.004
峰位置:	801.96	强度:	85.951
峰位置:	849.00	强度:	87.792
峰位置:	876.49	强度:	89.907
峰位置:	921.29	强度:	91.546
峰位置:	952.11	强度:	91.399
峰位置:	1044.56	强度:	77.244
峰位置:	1087.95	强度:	72.028
峰位置:	1127.02	强度:	75.998
峰位置:	1166.13	强度:	65.657
峰位置:	1284.62	强度:	61.904
峰位置:	1311.57	强度:	69.360
峰位置:	1384.30	强度:	62.790
峰位置:	1419.96	强度:	70.042
峰位置:	1446.84	强度:	69.981
峰位置:	1512.13	强度:	74.699
峰位置:	1607.94	强度:	46.798
峰位置:	1656.13	强度:	56.180
峰位置:	2931.82	强度:	84.468
峰位置:	2971.95	强度:	82.594
峰位置:	3427.65	强度:	39.160

Figure S1.2 IR spectrum of compound **1**

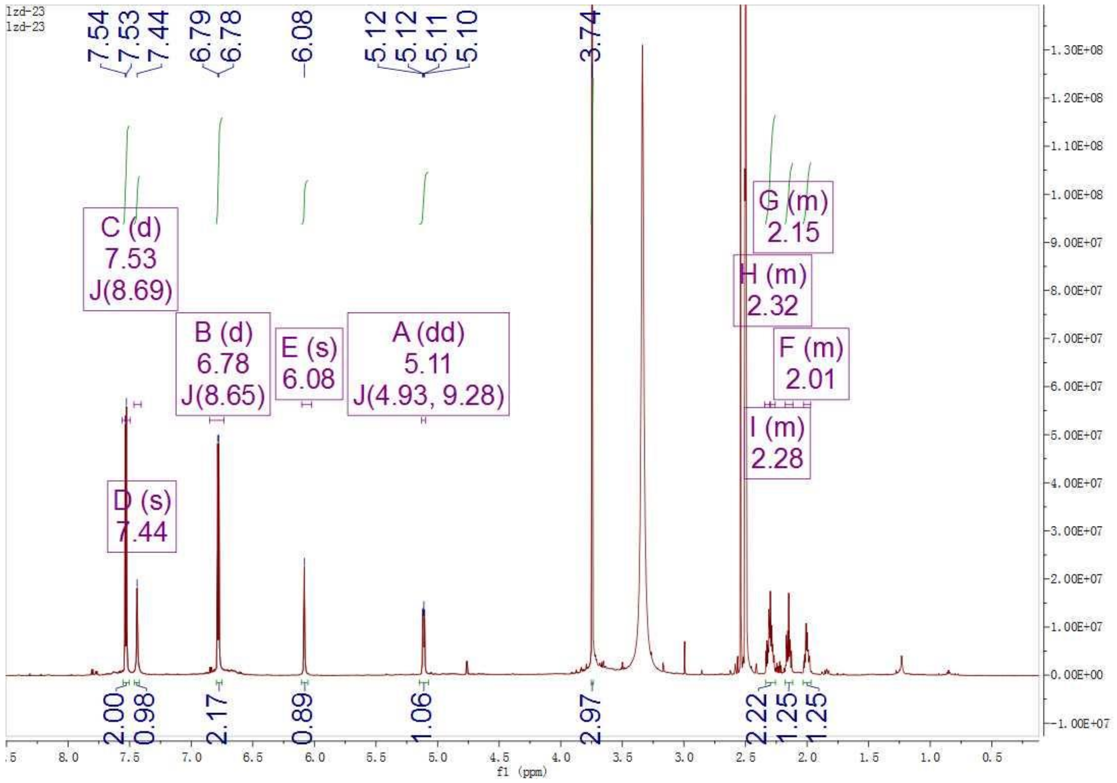

Figure S1.3 ^{1}H NMR spectrum (800 MHz, DMSO-d_6) of compound **1**

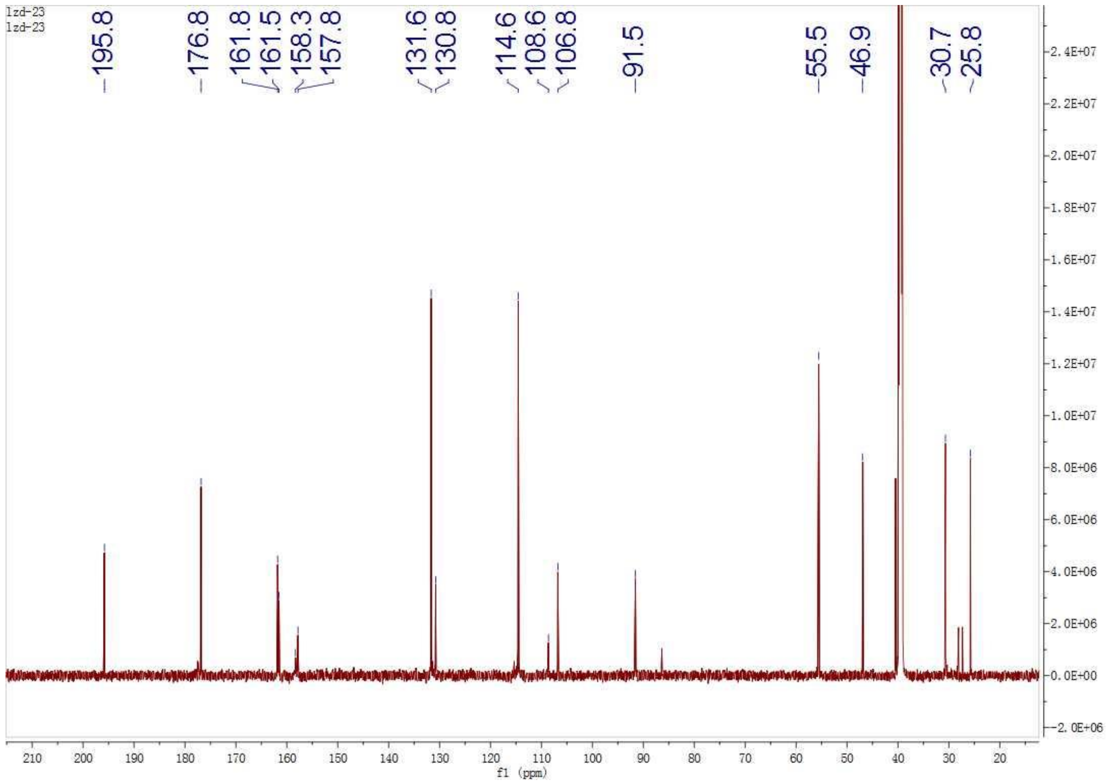

Figure S1.4 ^{13}C NMR spectrum (200 MHz, DMSO-d_6) of compound **1**

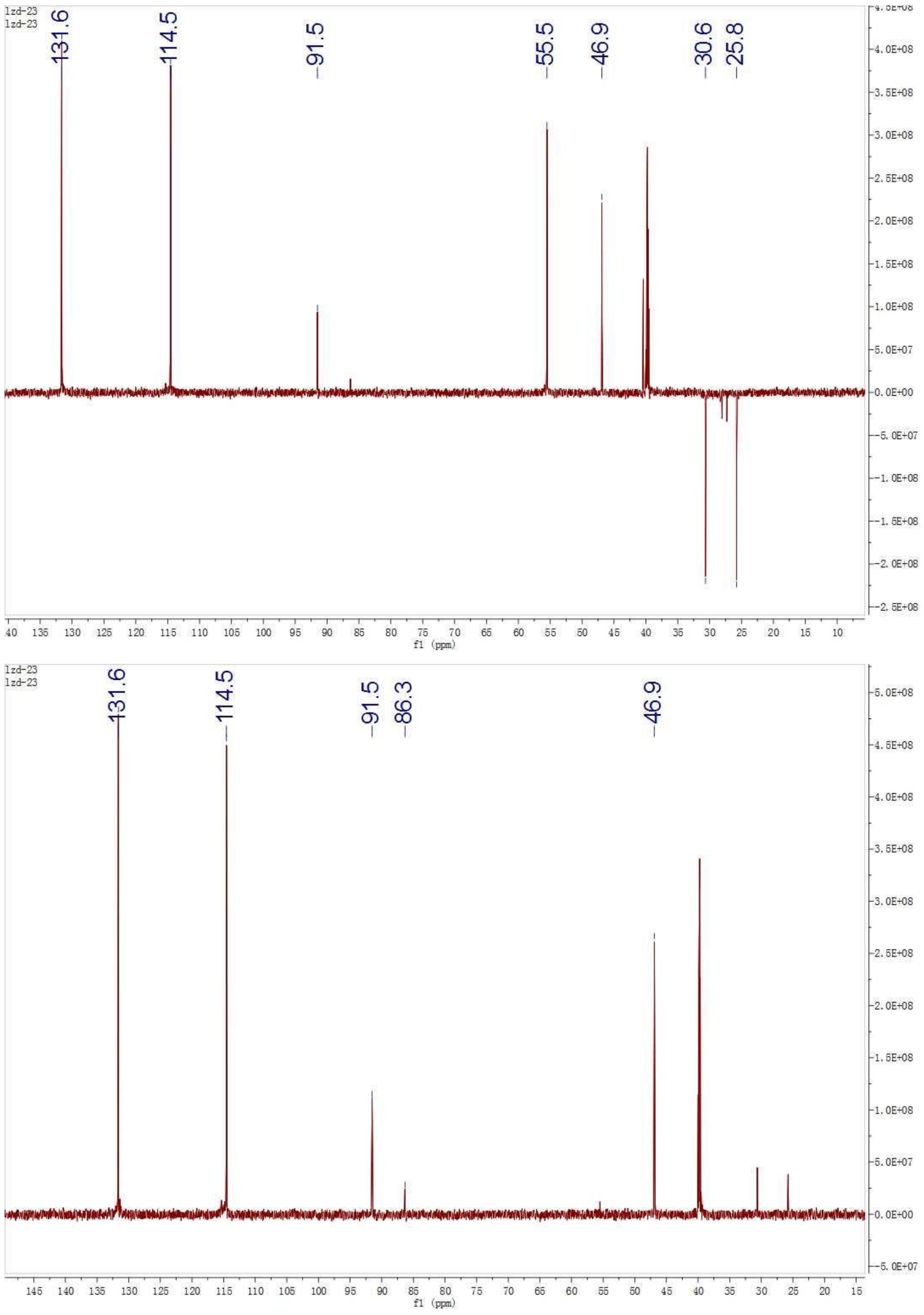

Figure S1.5 DEPT spectrum (200 MHz, DMSO-d_6) of compound **1**

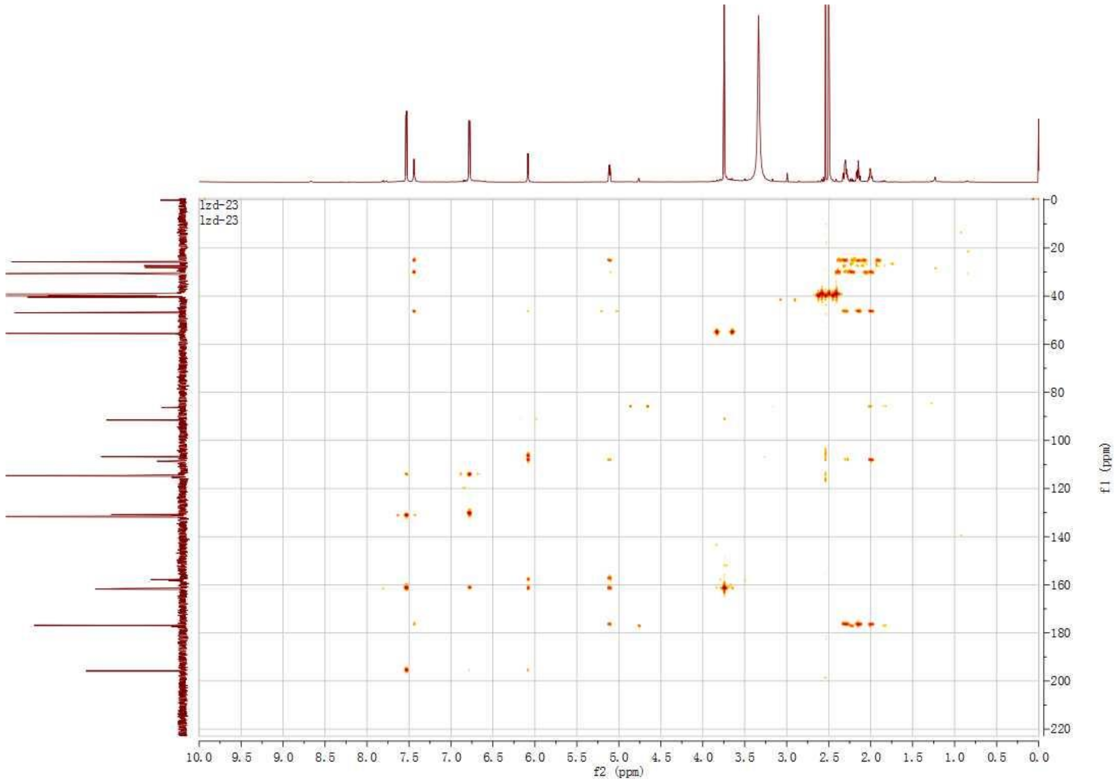

Figure S1.6 HMBC spectrum (800 MHz, DMSO-d_6) of compound **1**

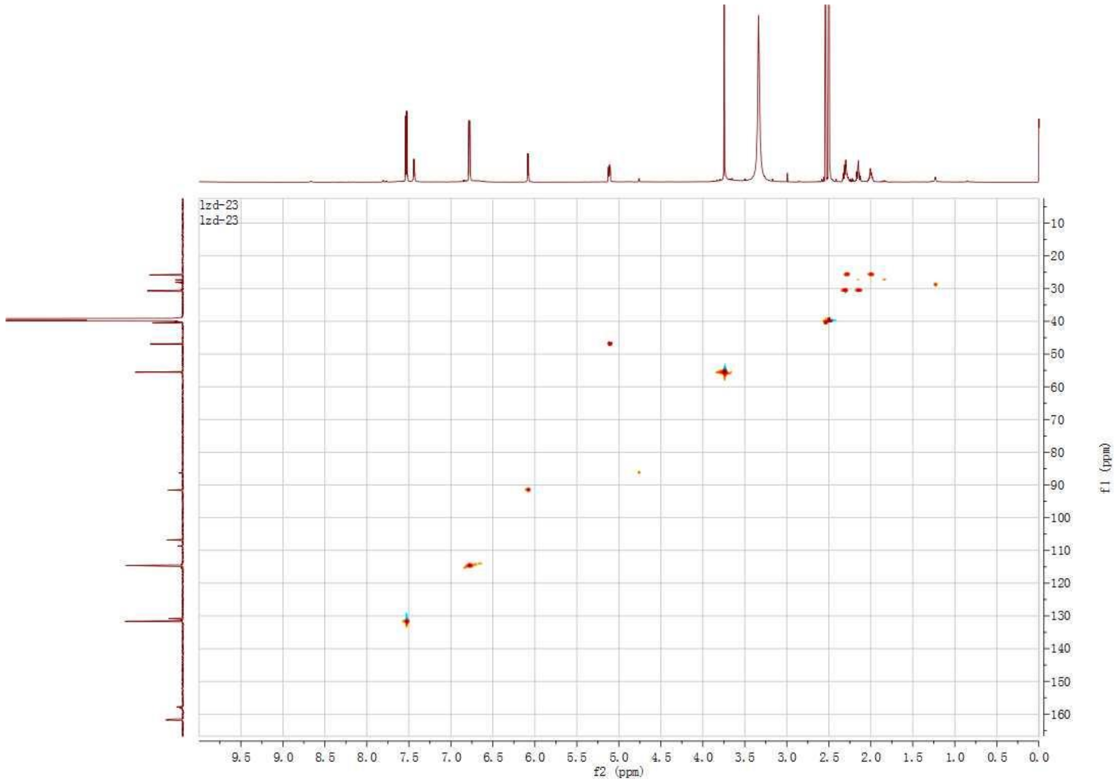

Figure S1.7 HSQC spectrum (800 MHz, DMSO-d_6) of compound **1**

Qualitative Analysis Report

**Data Filename**	190228ESIA7.d	**Sample Name**	zlzd-23
**Sample Type**	Sample	**Position**	
**Instrument Name**	Agilent G6230 TOF MS	**User Name**	KIB
**Acq Method**	ESI.m	**Acquired Time**	2/28/2019 10:31:36 AM
**IRM Calibration Status**	Success	**DA Method**	ESI.m
**Comment**			

Sample Group **Info.**

Acquisition SW Version 6200 series TOF/6500 series Q-TOF B.05.01 (B5125.2)

User Spectra

Fragmentor Voltage	Collision Energy	Ionization Mode
240	0	ESI

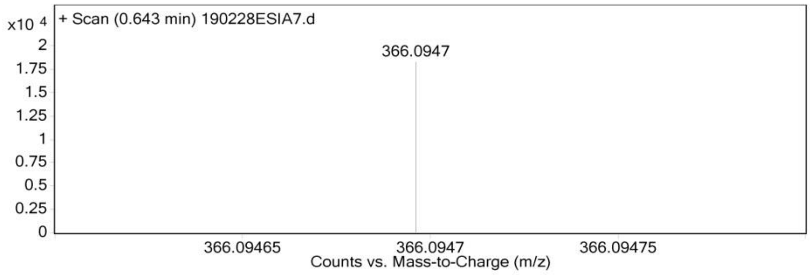

Peak List

m/z	z	Abund	Formula	Ion
121.0509	1	28154.41		
312.362	1	9971.65		
366.0947	1	18319.05	C18 H17 N Na O6	M+
367.1006	1	43828.62		
407.1217	1	11287.66		
408.1277	1	26718.01		
409.161	1	34730.88		
710.2071	1	17512.92		
711.2116	1	27672.05		
922.0098	1	29409.73		

Formula Calculator Element Limits

Element	Min	Max
C	0	200
H	0	400
O	0	10
Na	1	1
N	1	1

Formula Calculator Results

Formula	CalculatedMass	Mz	Diff.(mDa)	Diff. (ppm)	DBE
C18 H17 N Na O6	366.0954	366.0947	0.7	1.8	10.5

--- End Of Report ---

Figure S1.8 HRESIMS spectrum of compound **1**

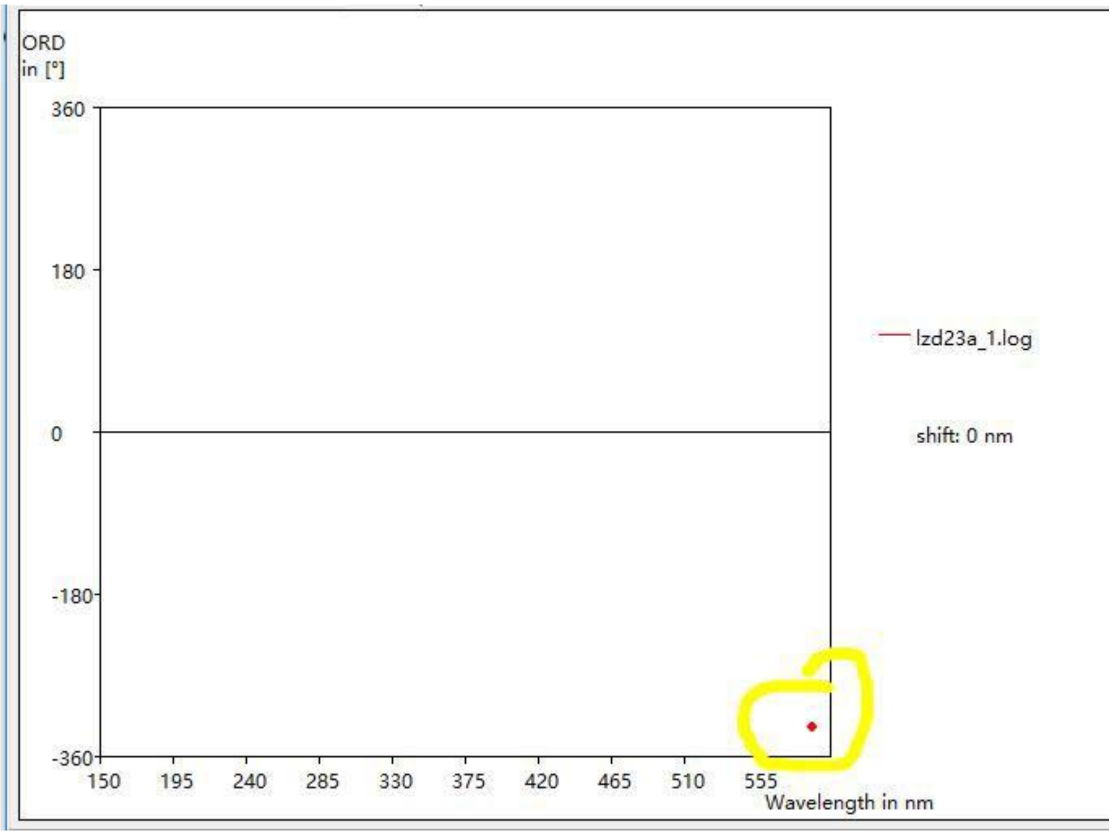


```
dimension    70 with     3 vectors. FullF1:  Do perturbations    1 to     3. Dipole-
magnetic dipole polarizability for W=    0.077318:                1              2
   3        1    0.154262D+02  0.164532D+02 -0.395992D+02       2    0.443034D+02 -0.960516D+02
-0.351420D+02       3   -0.401683D+03 -0.432994D+02  0.893424D+02 w=     0.077318 a.u.,
Optical Rotation Beta=      -2.9057 au. Molar Mass =    343.3354 grams/mole, [Alpha] (
5893.0 A) =       -327.12 deg. End of Minotr F.D. properties on file    721 Mask=       2
NFrqRd=     1 NDeriv=     1 ND12= 1 LenFil=      12: Frequencies=    0.077318 Property
number 2 -- FD Optical Rotation Tensor frequency  1    0.077318:                1
   2              3        1    0.154262D+02  0.164532D+02 -0.395992D+02       2    0.443034D+02
-0.960516D+02 -0.351420D+02       3  -0.401683D+03 -0.432994D+02  0.893424D+02 End of Minotr
F.D. properties file   722 does not exist. End of Minotr F.D. properties file   788 does
not exist. Leave Link 1002 at Wed Apr 17 21:11:08 2019, MaxMem=  4294967296 cpu:
34438.2 (Enter /soft/apps/Paid//g09/l601.exe) Copying SCF densities to generalized density
rwf, IOpCl= 0 IROHF=0.
**********************************************************************
```

$[\alpha]_{D}^{25}$ -327.12 (*c* 0.10, MeOH) (calculated OR)

Figure S1.9 Calculating OR spectra of compound **1**

Table S1 Growth inhibition of HepG2 and Hep3B cells in the presence of different compounds after 72 h incubation (IC50 values in nM).

compounds	Cells IC50 (nM)	
	HepG 2	Hep 3B
1	153.10±34.30**	180.6±31.63**
2	3161±207.55**	6250±358.93**
3	909.10±64.37**	1182±55.76**
4	1764±105.65**	2274±109.78**
5-Fluorouracil	5659±315.72	51709±370.67

All data were shown as means ± SD of three independent experiments. *$p < 0.05$, **$p < 0.01$ compared with positive control.
